# Calcium Hydroxyapatite Biostimulators: A Comparative Study of Biological Response and Particle Morphology

**DOI:** 10.3390/biomedicines14071447

**Published:** 2026-06-25

**Authors:** Valéria Dal Col, Bibiana Franzen Matte

**Affiliations:** 1 Protocoll Clinique, 721 Aleixo Neto Street, Vitória 29055-260, ES, Brazil; 2 Nucleo Vitro, Porto Alegre 91040-600, RS, Brazil; bibiana@nucleovitro.com

**Keywords:** calcium hydroxyapatite, CaHA, dermal biostimulators, human dermal fibroblasts, RT-qPCR, MTT assay, scanning electron microscopy

## Abstract

**Background/Objectives:** Calcium hydroxyapatite (CaHA)-based injectable materials are widely used as dermal biostimulators. In vitro models allow for controlled comparison of cellular responses and particle characteristics across formulations. This study aimed to compare two commercially available CaHA-based materials in terms of fibroblast metabolic activity, extracellular matrix-related gene expression, and microsphere morphology. **Methods:** Primary human dermal fibroblasts were exposed to two CaHA-based materials (Sample R and Sample S) at 10 mg/mL. Metabolic activity was assessed using the MTT assay at 24, 36, 48, and 72 h. Type I collagen and elastin gene expression were evaluated by RT-qPCR at 72 h. Microsphere morphology was analyzed by scanning electron microscopy (SEM). **Results:** Both materials increased fibroblast metabolic activity compared with the control at all time points. Early responses were similar, whereas Sample S showed higher activity at 48 and 72 h. At 72 h, both materials increased collagen and elastin gene expression versus the control, with greater responses observed for Sample S. SEM analysis showed predominantly spherical microspheres in both materials, with qualitative differences in surface microtopography. **Conclusions:** Under controlled in vitro conditions, both CaHA-based materials were biocompatible and modulated fibroblast metabolic activity and extracellular matrix-related gene expression. Differences in particle surface characteristics may contribute to the observed biological profiles. These findings support further studies incorporating extended incubation periods and protein-level analyses.

## 1. Introduction

The dermis is a specialized connective tissue composed predominantly of extracellular matrix (ECM) proteins, including collagen fibers, elastic fibers, and proteoglycans, which together confer mechanical strength, elasticity, and structural integrity to the skin. Dermal fibroblasts are the principal cells responsible for the synthesis, organization, and remodeling of these matrix components, and their behavior is central to dermal homeostasis [1,2].

Calcium hydroxyapatite (CaHA) is a calcium phosphate-based bioceramic with physicochemical characteristics that allow for interaction with connective tissue cells. As a biomaterial, CaHA has been extensively studied in nonclinical settings due to its biocompatibility, structural stability, and ability to act as a substrate for cellular interaction [3,4]. Beyond its chemical composition, the biological response to CaHA may be influenced by particle morphology, surface characteristics, and particle-specific properties [5,6,7]. Several in vitro and histological studies have demonstrated that CaHA-based materials can directly interact with dermal fibroblasts and ECM-associated structures, inducing measurable cellular responses and matrix-related changes [7,8,9].

Despite increasing evidence of CaHA–fibroblast interactions, direct and standardized side-by-side comparisons between commercially available CaHA-based materials remain limited. In particular, comparisons between products with similar nominal composition under identical experimental conditions are scarce.

Therefore, the present study provides a comparative in vitro evaluation of two commercially available CaHA-based injectable materials with similar compositional profiles. By integrating fibroblast metabolic activity, extracellular matrix-related gene expression, and microsphere morphology under standardized conditions, this study explores whether material-specific characteristics beyond nominal composition may be associated with differential biological responses.

## 2. Materials and Methods

This study comprises a comparative in vitro evaluation. Human dermal fibroblast responses to two CaHA-based injectable implants were assessed under controlled conditions, including metabolic activity and extracellular matrix-related gene expression. In parallel, CaHA microsphere morphology and surface features were characterized by scanning electron microscopy (SEM).

### 2.1. Cell Culture

Primary human dermal fibroblasts were obtained from a healthy donor under an Ethics Committee/IRB-approved protocol permitting secondary research use (CAAE #59124916.6.0000.5327) and were used for all in vitro assessments of fibroblast metabolic activity (MTT) and extracellular matrix (ECM)-related parameters. Cells were maintained in Dulbecco’s Modified Eagle’s Medium (DMEM, Gibco, Grand Island, NY, USA) supplemented with fetal bovine serum (FBS, Gibco, Grand Island, NY, USA) and routine additives according to the laboratory protocol. Cultures were incubated at 37 °C in a humidified atmosphere containing 5% CO_2_, and all handling was performed under sterile conditions in a laminar flow hood. Fibroblasts were seeded in standard culture plates at a density of 1 × 10^3^ cells/well.

#### 2.1.1. Sample Preparation and Exposure

The calcium hydroxyapatite (CaHA)-based injectable materials evaluated in this study were Radiesse, hereafter referred to as Sample R, and Stiim, hereafter referred to as Sample S. The main characteristics of both materials, according to manufacturer information and product documentation, are summarized in Table 1. Both products were commercially available, used within their expiration dates, stored according to the manufacturers’ recommendations until use, and handled under sterile conditions following standard laboratory practices.

Test samples were prepared at a standardized concentration of 10 mg/mL by diluting each product in the corresponding cell culture medium immediately before use. For treatment, 100 μL/well of the test solutions (Sample R or Sample S) was applied in direct contact with human dermal fibroblast cultures. Control wells received the same supplemented culture medium (DMEM with FBS) without the test material. All experimental conditions were performed in triplicate. Following exposure, cultures were maintained at 37 °C in a humidified atmosphere containing 5% CO_2_ for up to 72 h, according to the predefined experimental time points.

#### 2.1.2. MTT Assay of Fibroblast Metabolic Activity

Fibroblast metabolic activity was evaluated at 24, 36, 48, and 72 h using the 3-(4,5-dimethylthiazol-2-yl)-2,5-diphenyltetrazolium bromide (MTT) assay. At each time point, the culture medium was removed and cells were incubated with MTT solution(Sigma-Aldrich, St. Louis, MO, USA) (1 mg/mL) for 2 h at 37 °C in a humidified atmosphere containing 5% CO_2_. The MTT solution was then discarded, and isopropanol (Sigma-Aldrich, St. Louis, MO, USA) was added to solubilize formazan crystals (30 min). Absorbance was measured at 570 nm using a microplate reader. The control group (supplemented medium only) was normalized to 100%, and the results for Sample R and Sample S were expressed as percent increases relative to normalized control. All conditions were performed in triplicate.

#### 2.1.3. Gene Expression Analysis (RT-qPCR)

Total RNA was extracted from human dermal fibroblast cultures after 72 h of exposure to Sample R, Sample S, or supplemented medium (control) using Trizol reagent (Thermo Fisher Scientific, Waltham, MA, USA), and RNA purity was assessed by spectrophotometry (A260/A280 ratio 1.8–2.0). Complementary DNA (cDNA) was synthesized from 2000 ng of total RNA using a high-capacity reverse transcription kit (Thermo Fisher Scientific, Waltham, MA, USA), following the manufacturer’s instructions.

Quantitative real-time PCR (RT-qPCR) was performed using SYBR Green chemistry (Thermo Fisher Scientific, Waltham, MA, USA) to evaluate type I collagen and elastin expression, with β-actin as the endogenous control. Primer sequences were as follows: type I collagen (forward AGGGCCAAGACATC; reverse AGATCACGTCATCGCACAACA), elastin (forward GTATATACCCAGGTGGAGTG; reverse CGAACTTTGCTGCTGCTTTAG), and β-actin (forward CCAGAGGCGTAGAGGGATAG; reverse CCAACCGCGAGAAGATGA). Cycling conditions were: 95 °C for 10 min, followed by 40 cycles of 95 °C for 15 s, 60 °C for 30 s, and 75 °C for 15 s, with a final extension at 75 °C for 10 min. Relative gene expression was calculated using the 2^−ΔΔCt^ method and expressed as fold-change versus the control.

#### 2.1.4. Statistical Analysis

All quantitative data were compiled and processed using Microsoft Excel and GraphPad Prism version 9.0 (GraphPad Software, San Diego, CA, USA). The results were expressed as mean ± standard deviation (SD) from triplicate measurements (n = 3), as reported by the external laboratory. Comparisons among groups (Sample R, Sample S, and control) were performed using one-way analysis of variance (ANOVA) followed by Bonferroni’s multiple-comparisons post hoc test. Statistical significance was defined as *p* < 0.05.

### 2.2. Scanning Electron Microscopy (SEM) Particle Characterization

CaHA microspheres were isolated following a previously described washing/centrifugation protocol for CaHA-based fillers [9,10]. Briefly, 1 mL of the CaHA solution was diluted in 10 mL of Milli-Q water and vortexed for 5 min. Microspheres were then sedimented by centrifugation at 5000 rpm for 5 min. This washing cycle was repeated until no visible residues of the polymeric gel carrier remained. The recovered microspheres were dried and transferred onto carbon tape mounted on SEM stubs; excess particles were removed by gentle nitrogen blowing. Micrographs were obtained at multiple magnifications for qualitative assessment of particle geometry and surface features. Particle size measurements and histogram-based size distribution analyses were not performed. SEM was used for qualitative morphological characterization of particle geometry and surface features.

## 3. Results

### 3.1. Fibroblast Metabolic Activity (MTT)

**Figure 1:**
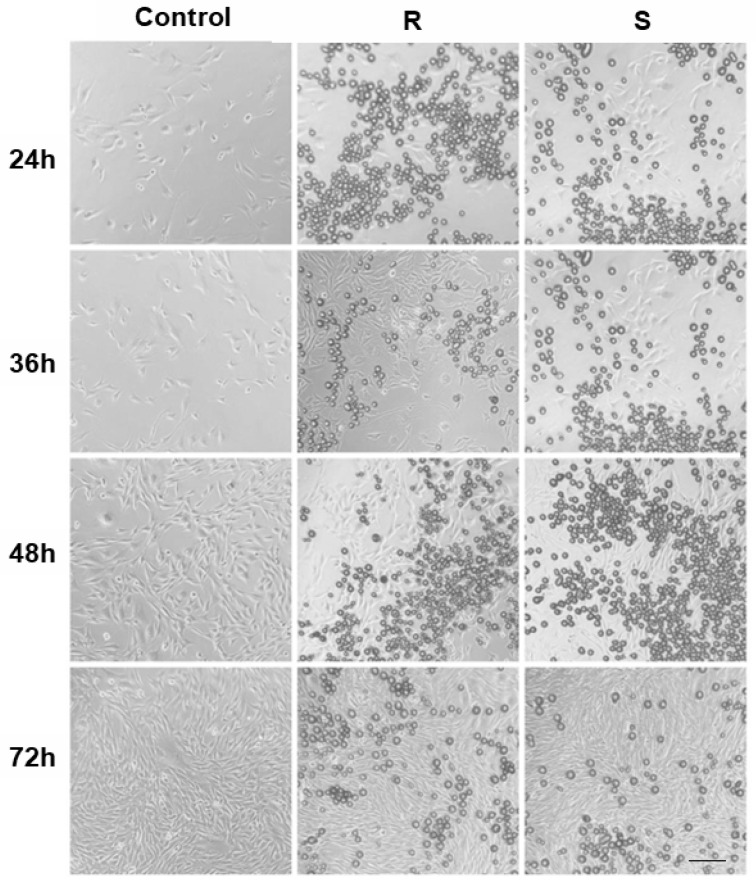
Representative images of human dermal fibroblast cultures at 24, 36, 48, and 72 h. Images show cellular presence in the control group and after exposure to samples. Scale bar = 100 µm.

**Figure 2:**
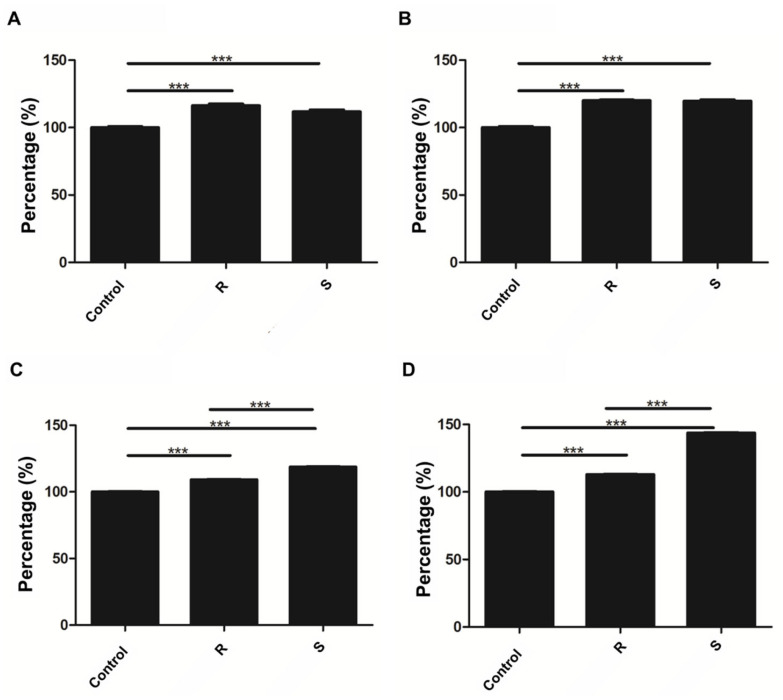
MTT assay of fibroblast metabolic activity. (**A**) 24 h; (**B**) 36 h; (**C**) 48 h; (**D**) 72 h. The results are expressed as percentage relative to the control group, which was normalized to 100%. Values above 100% indicate increases relative to the normalized control. Data are presented as mean ± SD (n = 3). *** *p* <0.001.

Fibroblasts were seeded and treated in standard culture plates at 24, 36, 48, and 72 h (Figure 1) and fibroblast viability/metabolic activity was assessed by MTT. Both Sample R and Sample S increased viability versus the control at all time points (*p* < 0.001). At 24 h and 36 h, the responses between the samples were not statistically significant. From 48 h onward, Sample S showed a greater increase above the normalized control than Sample R (48 h: +18.7 ± 0.4% vs. +9.1 ± 0.4%; 72 h: +43.6 ± 0.31% vs. +12.8 ± 0.31%; *p* < 0.001 for between-sample comparisons at 48 and 72 h) (Figure 2).

### 3.2. Collagen and Elastin Gene Expression

**Figure 3:**
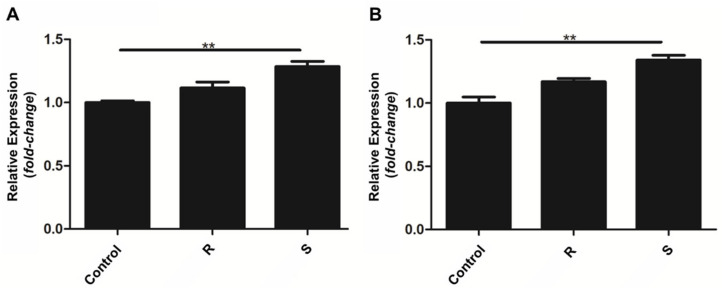
Relative gene expression of type I collagen and elastin after 72 h of exposure. (**A**) Type I collagen gene expression relative to control cultures (normalized to 1.0). Sample R showed a modest increase compared with the control that did not reach statistical significance, whereas Sample S demonstrated a significant increase versus the control (** *p* < 0.01). (**B**) Elastin gene expression relative to control cultures. Sample R showed an increase compared with the control without statistical significance, while Sample S exhibited a significant increase compared with the control (** *p* < 0.01). Data are presented as mean ± SD. Statistical significance is indicated relative to the control group.

After 72 h of exposure, type I collagen and elastin gene expression were assessed relative to control cultures. For type I collagen, the control group was normalized to 1.0 (±0.025). Sample R showed an 11.6% (±8.2%) increase compared with the control; however, this difference did not reach statistical significance. In contrast, Sample S showed a 22.9% (±6.9%) increase versus the control, which was statistically significant (*p* < 0.01, Figure 3). Sample S exhibited numerically higher collagen expression than Sample R.

For elastin expression, Sample R increased by 17.0% (±4.4%) compared with the control, but this increase was not statistically significant. Conversely, Sample S showed a 34.0% (±6.6%) increase versus the control, reaching statistical significance (*p* < 0.01, Figure 3). Sample S also exhibited numerically higher elastin expression than Sample R.

### 3.3. SEM Particle Morphology and Surface Features

**Figure 4:**
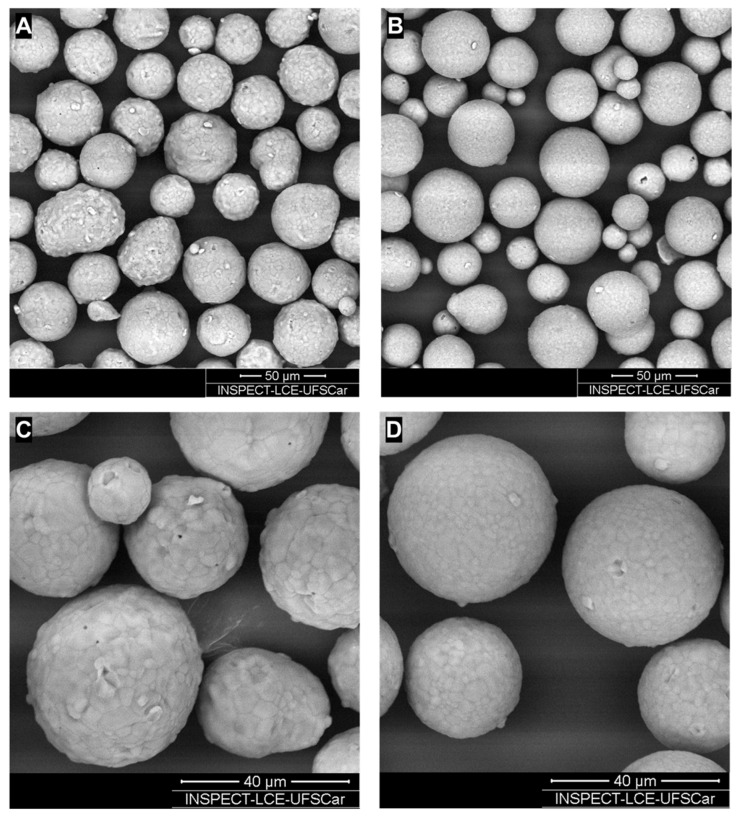
SEM comparison of CaHA particle morphology. (**A**) Sample R, 1000×; (**B**) Sample S, 1000×; (**C**) Sample R, 2000×; (**D**) Sample S, 2000×. Representative scanning electron microscopy (SEM) micrographs of CaHA particles. Scale bars are shown in the images.

Scanning electron microscopy (SEM) was performed to evaluate the morphology and surface architecture of the microparticles from both products. Representative micrographs are shown in Figure 4. Both products exhibited predominantly spherical microparticles with apparent variability in particle dimensions. The particles appeared well-defined, with no evidence of significant fragmentation or irregular debris.

Despite the overall similarity in particle geometry, differences in surface morphology were observed between the two products. Microparticles from Sample R (Figure 4A,C) exhibited a more irregular and microgranular surface with evident nodular features and surface heterogeneity. In contrast, microparticles from Sample S (Figure 4B,D) showed a comparatively smoother and more homogeneous surface architecture, while maintaining the spherical particle morphology.

Higher-magnification images (Figure 4C,D) further highlighted these surface characteristics. Sample R particles displayed a pronounced microtextured surface with irregular microdomains distributed across the particle surface. In comparison, sample S particles exhibited a more uniform surface microstructure with reduced surface irregularities.

## 4. Discussion

The present study integrates data from two complementary in vitro analyses to compare the biological response of human dermal fibroblasts exposed to two calcium hydroxyapatite (CaHA)-based injectable materials. By employing the experimental design of controlled cell culture conditions, the analysis focused directly on material–cell interactions, independent of systemic, vascular, or immune influences.

Both samples increased fibroblast metabolic activity relative to control conditions across all evaluated time points, supporting their biocompatibility under the tested experimental parameters.

In vitro metabolic assays such as MTT are widely used in biomaterials research as indicators of cellular compatibility and metabolic responsiveness following exposure to foreign materials [1,11]. Although early responses were comparable between the samples, Sample S demonstrated higher metabolic activity at 48 and 72 h, suggesting a formulation-dependent divergence during sustained exposure.

In parallel, collagen- and elastin-related gene expression at 72 h increased for both materials compared with control cultures, with higher responses observed for Sample S. Together, these results indicate that both CaHA formulations can modulate fibroblast activity and extracellular matrix-related endpoints in vitro, with magnitude varying between products.

These findings are consistent with previous reports demonstrating that CaHA-based materials can stimulate fibroblast activity and promote extracellular matrix remodeling, particularly through direct particle–cell contact mechanisms [8]. In vitro studies have shown increased collagen and elastin expression following CaHA exposure, supporting its well-established biostimulatory profile [8,12,13,14,15]. Additionally, variations in particle morphology, size, and surface characteristics across commercially available CaHA-based products have been described, which may influence their biological behavior and interaction with surrounding tissue [16,17].

Although both materials share a similar CaHA-based composition, differences in biological response were observed under identical experimental conditions. These findings suggest that factors beyond nominal composition, such as microsphere surface characteristics and microtopography, may contribute to the modulation of fibroblast behavior.

SEM characterization showed that both products consisted predominantly of spherical CaHA microspheres, consistent with previously reported structural features of CaHA-based dermal biostimulators. However, qualitative differences in particle surface microtopography were observed between the products. In biomaterial science, surface topography and 3D scaffold architecture are known to influence cell adhesion, mechanotransduction, and cell–material interactions, thereby possibly influencing fibroblast behavior and extracellular matrix response over time [6,18]. Particle size, morphology, and distribution are known to influence biomaterial–cell interactions. In this study, both manufacturers report a similar particle size range (25–45 μm), and the analysis therefore focused on morphological features and surface microtopography. Quantitative evaluation of particle size distribution, including histogram-based analysis, was not performed and represents a limitation of this study. X-ray diffraction (XRD) analysis was not performed in the present study; therefore, independent confirmation of the crystalline phase composition of the CaHA microspheres was beyond the scope of this work. Future studies should incorporate XRD or complementary physicochemical analyses to further characterize the phase composition of CaHA-based injectable materials. In this context, SEM observations of surface differences between the materials provide morphological information that may be associated with the distinct in vitro profiles, although causal relationships cannot be established within the scope of the present study.

Building on these observations, the results should be interpreted within the constraints of an in vitro model. ECM-related outcomes were assessed at the gene-expression level, and metabolic activity was evaluated using MTT assays; these parameters may not directly translate to protein synthesis, matrix deposition, or tissue-level remodeling. Furthermore, no protein-level or extracellular matrix deposition assays were performed, and inflammatory/immune-cell readouts were not evaluated. Consequently, the model does not capture the full biological complexity of native dermal tissue, and biostimulatory effects should be interpreted with caution. These constraints include the use of a single cell type, one exposure concentration, and a relatively short incubation period. Future studies incorporating dose variation, longer exposure times, and more complex systems, such as 3D or co-culture models, may provide a more comprehensive understanding of material–cell interactions.

Despite these limitations, this study provides a concise, standardized side-by-side comparison integrating fibroblast metabolic activity, ECM-related gene expression, and SEM particle morphology. Such standardized nonclinical models may contribute to the understanding of biomaterial–cell interactions and serve as a foundation for further investigations incorporating longer incubation periods, protein-level analyses, and immune-relevant endpoints.

## 5. Conclusions

Both CaHA-based materials demonstrated compatibility with human dermal fibroblast cultures under the tested in vitro conditions and were associated with increased fibroblast metabolic activity and extracellular matrix-related gene expression compared with control cultures. Sample S showed higher metabolic activity and gene expression responses at later time points.

SEM analysis confirmed that both products consist of spherical CaHA microspheres while exhibiting differences in surface microtopography. These structural characteristics may influence material–cell interactions and contribute to the distinct in vitro profiles observed.

Overall, the findings provide a controlled comparative assessment of two CaHA-based dermal biostimulators and highlight the relevance of integrating biological and particle-level analyses in future nonclinical studies.

## Figures and Tables

**Table 1:** Main characteristics of the CaHA-based injectable materials evaluated in this study.

Characteristic	Sample R	Sample S
Commercial product	Radiesse Duo	Stiim
Manufacturer	Merz North America, Inc., Franksville, WI, USA	CG Bio Co., Ltd., Seongnam-si, Republic of Korea
Lot number	A00088880	S2Q23013
Material class	CaHA-based injectable implant	CaHA-based injectable implant
CaHA content	Approximately 30% CaHA by volume	Approximately 30% CaHA by volume
Carrier gel composition	Sterile water for injection, glycerin, and sodium carboxymethylcellulose	Water for injection, glycerin, and sodium carboxymethylcellulose
Reported CaHA particle size	25–45 µm	25–45 µm
Storage conditions	15–32 °C	15–25 °C
Exposure concentration in this study	10 mg/mL	10 mg/mL

## Data Availability

All data generated or analyzed during this study are included in this published article.
